# Diversity and distribution of *Wolbachia* in relation to geography, host plant affiliation and life cycle of a heterogonic gall wasp

**DOI:** 10.1186/s12862-018-1151-z

**Published:** 2018-03-27

**Authors:** Hannes Schuler, Scott P. Egan, Glen R. Hood, Robert W. Busbee, Amanda L. Driscoe, James R. Ott

**Affiliations:** 1 Institute of Forest Entomology, Forest Pathology and Forest Protection, Boku, University of Natural Resources & Life Sciences, Peter-Jordan-Straße 82/I, 1190 Vienna, Austria; 2 Present Address: Laimburg Research Centre, Laimburg 6, 39040 Pfatten, Italy; 3 Department of BioSciences, Rice University, Houston, TX 77005 USA; 4 Population and Conservation Biology Program, Department of Biology, Texas State University, San Marcos, TX 78666 USA

**Keywords:** Cynipidae, Endosymbiont, Horizontal transmission, Speciation, *wsp*

## Abstract

**Background:**

The maternally inherited endosymbiont *Wolbachia* is widespread in arthropods and nematodes and can play an important role in the ecology and evolution of its host through reproductive manipulation. Here, we survey *Wolbachia* in *Belonocnema treatae,* a widely distributed North American cynipid gall forming wasp that exhibits regional host specialization on three species of oaks and alternation of sexually and asexually reproducing generations. We investigated whether patterns of *Wolbachia* infection and diversity in *B. treatae* are associated with the insect’s geographic distribution, host plant association, life cycle, and mitochondrial evolutionary history.

**Results:**

Screening of 463 individuals from 23 populations including sexual and asexual generations from all three host plants across the southern U.S. showed an average infection rate of 56% with three common *Wolbachia* strains: *w*Tre1–3 and an additional rare variant *w*Tre4. Phylogenetic analysis based on *wsp* showed that these strains are unrelated and likely independently inherited. We found no difference in *Wolbachia* infection frequency among host plant associated populations or between the asexual and sexual generations, or between males and females of the sexual generation. Partially incomplete *Wolbachia* transmission rates might explain the occurrence of uninfected individuals. A parallel analysis of the mitochondrial cytochrome oxidase I gene in *B. treatae* showed high mtDNA haplotype diversity in both infected and uninfected populations suggesting an ancestral infection by *Wolbachia* as well as a clear split between eastern and western *B. treatae* mtDNA clades with a sequence divergence of > 6%. The strain *w*Tre1 was present almost exclusively in the western clade while *w*Tre2 and *w*Tre3 occur almost exclusively in eastern populations. In contrast, the same strains co-occur as double-infections in Georgia and triple-infections in two populations in central Florida.

**Conclusions:**

The diversity of *Wolbachia* across geographically and genetically distinct populations of *B. treatae* and the co-occurrence of the same strains within three populations highlights the complex infection dynamics in this system. Moreover, the association of distinct *Wolbachia* strains with mitochondrial haplotypes of its host in populations infected by different *Wolbachia* strains suggests a potential role of the endosymbiont in reproductive isolation in *B. treatae.*

**Electronic supplementary material:**

The online version of this article (10.1186/s12862-018-1151-z) contains supplementary material, which is available to authorized users.

## Background

*Wolbachia* are maternally inherited intracellular bacteria reported in a diverse range of arthropod species worldwide (reviewed in [1]). This endosymbiont has wide reaching impacts on the biology of its host [2], including effects on survival through immunity and nutrition [3, 4], and reproduction through male killing, feminization, parthenogenesis, and cytoplasmic incompatibility [2, 5]. In this regard, manipulation of host reproduction by *Wolbachia* is an important consideration in plant-insect systems and often plays an important role in a variety of evolutionary processes, including local adaptation, gene flow, host-associated differentiation, and speciation [6, 7].

In particular, *Wolbachia* induced cytoplasmic incompatibility (CI) has important evolutionary implications for insect populations distributed across space and differing ecological environments. Cytoplasmic incompatibility results in male and female gametes being unable to form viable offspring due to differences in parental *Wolbachia* infection status. When *Wolbachia* infected males mate with uninfected females (unidirectional infection) few or no offspring are produced while all other crosses are fertile [8]. As a result, once infection levels in a population surpass a threshold, *Wolbachia* is predicted to sweep through the host population [9–11]. The reproductive advantage of *Wolbachia* infected individuals can result in rapid spread of the endosymbiont [9, 12]. Moreover, the co-maternally inherited mitochondrial DNA can hitchhike with the spreading *Wolbachia* replacing mitochondrial haplotypes associated with uninfected individuals [11, 13]. Thus, *Wolbachia* infected populations typically exhibit lower mitochondrial diversity than uninfected populations [13–15]. *Wolbachia* infected populations are also usually associated with specific mitochondrial haplotypes i.e., those of their founder individuals which acquired *Wolbachia* horizontally from other hosts [12] or due to hybrid introgression [16].

When both partners are infected with different strains of *Wolbachia,* bidirectional CI occurs with both mating directions being infertile. This has peaked interest in the possible role of *Wolbachia* in promoting speciation or maintaining species boundaries [6, 7, 17, 18]. In essence, bidirectional CI can increase reproductive isolation by decreasing gene flow among populations that harbor different *Wolbachia* strains [19]. The presence of CI can also produce selection for other pre-zygotic isolating mechanisms. For example, *Wolbachia* induces behavioral isolation in mushroom feeding *Drosophila* via selection against heterospecific incompatible matings of uninfected females with infected males [20]. Therefore, the endosymbiont may promote divergence even within species that are infected by only one *Wolbachia* strain. Further, different *Wolbachia* strains promote pre- and postzygotic isolation in the *Drosophila paulistorum* species complex where the symbiont acts as a pathogen in hybrid males causing both embryonic inviability and male sterility [21]. Bidirectional CI is the primary form of reproductive isolation between species of *Nasonia* wasps: *Nasonia giraulti* and *Nasonia longicornis* harbor different *Wolbachia* strains and are reproductively isolated [19].

**Fig. 1:**
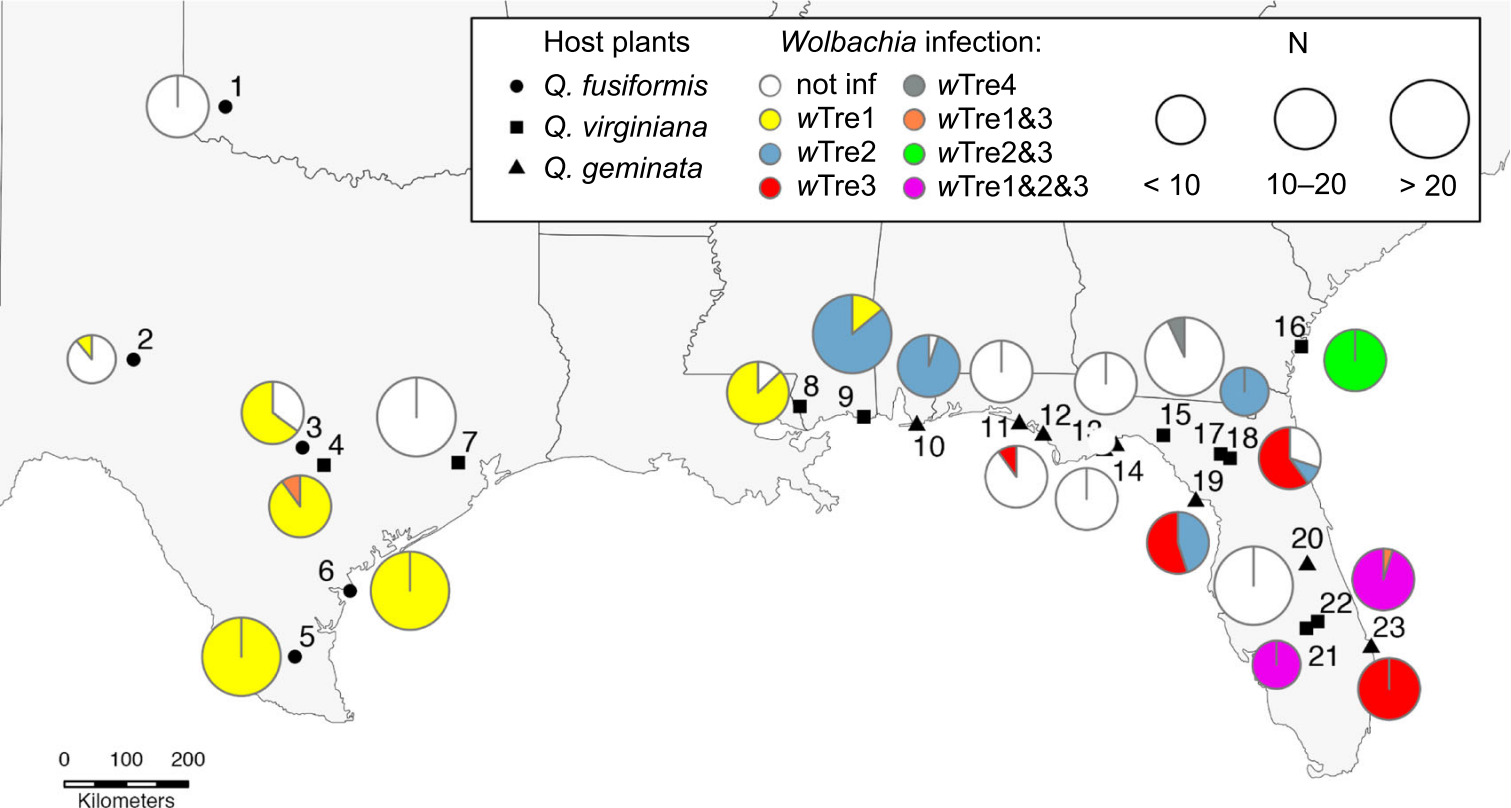
*Wolbachia* infection frequencies in each of 23 *Belonocnema treatae* populations distributed across the southern United States and sampled from three host plant species. Filled circles = *Quercus fusiformis*, filled squares = *Quercus virginiana* and filled triangles = *Quercus geminata.* Pie charts represent the percentage of *B. treatae* infected by *Wolbachia* in each population: white = uninfected individuals, yellow = *w*Tre1, blue = *w*Tre2, red = *w*Tre3, grey = *w*Tre4, orange = *w*Tre1&3, green = *w*Tre2&3, and purple = *w*Tre1&2&3. Sample size per population is denoted by size of the pie diagram. Details of population localities, sample size, sample composition and infection frequencies are given in Table 1. Map adapted from Wikimedia commons (CC BY-SA 3.0)

Given the wide-ranging effects of *Wolbachia* infection in generating and/or maintaining barriers to gene flow among populations, and our prior knowledge of the ecology and biology of the gall-forming wasp, *Belonocnema treatae*, Mayr (Hymenoptera: Cynipini: Cynipidae) we surveyed the infection status of host-associated populations of this cynipid. This species is a regional host specialist on closely related live oak species (genus *Quercus*; subsection *Virentes*) that are distributed across the southeastern U.S.: *Q. fusiformis* (*Qf*), *Q. virginiana* (*Qv*) and *Q. geminata* (*Qg*) (Fig. 1). Populations of *B. treatae* on *Qv* and *Qg* exhibit both partial positive assortative mating and host-associated habitat preference, and express significant differences in adult and gall morphology [22–24] in areas where the two host plants overlap. However, pairwise comparisons of neutral mtDNA markers among *B. treatae* populations resident on *Qv* and *Qg* across Florida revealed that sequence divergence is not associated with host use, as allopatric populations on the same host exhibited similar patterns of divergence compared to allopatric populations on different hosts [22]. Thus, these populations of *B. treatae* appear to be at the incipient stage of adaptive differentiation and speciation. What is currently not known is whether *Wolbachia* infection and/or strain diversity is involved in the partial reproductive isolation that is observed between different host-associated populations of *B. treatae* and whether *Wolbachia* is associated with potential population genetic structure of *B. treatae* evident on larger scales (i.e., across the geographic range of *B. treatae* in the southern U.S.).

The life cycle of *B. treatae* includes temporally and spatially isolated sexual and asexual generations (i.e., cyclical parthenogenesis or heterogony), common among cynipid gall wasps [25]. *Wolbachia*’s role as a sex ratio distorter [2] suggests a potential contribution of this endosymbiont in life cycle diversity of *B. treatae*. For example, Plantard et al. [26] demonstrated geographic variation in the prevalence of *Wolbachia* infection in the rose gall wasp *Diplolepsis spinosissimae* and showed that the endosymbiont is associated with obligate homozygous parthenogenesis. However, geographic variation in the prevalence of *Wolbachia* infection, infection with phylogenetic different *Wolbachia* strains, and the occasional occurrence of males highlights the complex infection history in this gall wasp species [26, 27]. Wachi et al. [28] concluded that *Wolbachia* induced CI may have led to the evolution of reproductive isolation between closely related oak gall wasps and thus contributed to the evolution of its host. In contrast, Abe et al. [29] described the occurrence of *Wolbachia* in the *Andricus mukaigawae* complex in Japan but did not detect an association of *Wolbachia* in bivoltine heterogonic and univoltine thelytokous species, thus excluding a potential role of the endosymbiont in the life cycle of this species complex. Screening of 64 gall wasp species by Rokas et al. [30] showed that *Wolbachia* is rare in oak gall wasps with just five species infected: three cyclical parthenogens (exhibiting both sexual and asexual generations) and two thelytokous species (exhibiting only the asexual generation). The authors excluded *Wolbachia* as a causative agent of life cycle diversity within the Cynipidae [30]. Although, this study found variable infection rates among species and populations within species, in some cases the limited number of samples examined (single individuals sampled per population, or single populations per species) may underestimate the number of species infected by the endosymbiont and thus might have underestimated the potential role of *Wolbachia* in the evolution of cynipids.

Our goal is to understand the ecological and evolutionary patterns and consequences of *Wolbachia* infection within and among populations of *B. treatae*. Herein, we address seven questions: (1) Given the large geographic range of *B. treatae*, we examine whether geographically separated populations within each host plant species display variable infection rates by the same or different *Wolbachia* strains? (2) Given that host-associated *B. treatae* populations exhibit partial reproductive isolation [22, 23], we ask whether populations associated with alternative host plant species differ in infection status or harbor different *Wolbachia* strains? (3) Given the generational differences in reproductive mode (parthenogenetic versus sexual reproduction), the presence of haploid males and diploid sexual females, development on different plant parts and at different times, and dramatic differences in the diversity and mortality attributed to insect parasitoids and inquilines [31, 32], we ask whether infection status differs between generations and between sexual males and females? (4) Given the prediction of reduced mitochondrial diversity due to the potential for selective sweeps of *Wolbachia* within infected populations, we inspect the relationship between *Wolbachia* infection and mitochondrial diversity of its host. (5) From the perspective of potential coevolution with the host, we ask whether the maternally inherited endosymbiont is associated with specific mitochondrial haplotypes of the host? (6) We ask, what is the phylogenetic relationship among *Wolbachia* strains resident in *B. treatae*? If closely related *Wolbachia* strains that are detected within *B. treatae* form a clade, this would suggest a deeper long-term association of the symbiont with *B. treatae*. In contrast, if multiple unrelated *Wolbachia* strains are present and distributed through the tree, repeated acquisition of *Wolbachia* by different gall wasp populations is implicated. (7) Finally, to further understand sources of among population variation in infection rates, we address the vertical transmission rate of a *Wolbachia* strain. Incomplete maternal transmission rates might explain different infection frequencies and potentially occasional loss of the endosymbiont.

To address these questions, we (1) sampled asexual and/or sexual generations of *B. treatae* from 23 populations distributed across a ~ 2400 km transect that spans the southern U.S. from all three host plants, (2) screened individuals for *Wolbachia* infection and examined strain diversity by sequencing a fragment of the *Wolbachia* surface protein (*wsp*) gene of all infected individuals, (3) sequenced a fragment of the mitochondrial cytochrome oxidase I gene of *B. treatae* to construct a phylogeny of *B. treatae* populations and (4) screened parents and offspring from single pair crosses to estimate vertical transmission rates of *Wolbachia*. We then mapped *Wolbachia* infection status and strain diversity across generation, geography, host plant affiliation and *B. treatae* phylogeography. Collectively, our results highlight a long evolutionary history of *Wolbachia* in *B. treatae* with complex infection dynamics across the geography, ecology, and life history of its host.

## Methods

### Study system

Gall wasps, family Cynipidae, comprise > 1300 species of secondarily phytophagous parasitic Hymenoptera [33–35]. Many cynipids, including *B. treatae* [36], exhibit heterogony (cyclical parthenogenesis), in which temporally and spatially segregated sexual and asexual generations alternate to complete the life cycle [25, 37]. The three species of host plants used by *B. treatae* in the southern U.S. exhibit partially overlapping geographic distributions (Fig. 1; [24, 38, 39]). Sexual generation *B. treatae* develop within multi-chambered root galls during the winter and emerge in the spring. Following mating, sexual females oviposit into the undersides of new leaves distributed throughout tree crowns and induce galls that each house a single asexual gall wasp. Asexual generation *B. treatae* consist of androphores and gynophores, which typically produce all male or all female sexual generation broods within root galls. Each root gall typically contains siblings produced by a single asexual female. Therefore, when surveying for *Wolbachia* (see below) within the sexual generation we screened only one individual from each root gall to avoid biased estimates of infection. The two generations do not overlap in time, exploit very different environments and are exposed to differing natural enemy communities. Asexual generation *B. treatae* developing in leaf galls in the crowns of trees are attacked by at least 20 species of insect natural enemies (parasitoids and inquilines) while the underground sexual generation developing in root galls are attacked by only 4 species [32]. Moreover, the percent of asexual leaf galls in which parasitoids and inquilines develop is far greater than in root galls housing the sexual generation. These spatial and temporal differences in the timing of galler development [31] and dramatic differences in natural enemy pressure [32] are possible sources of differential acquisition of *Wolbachia* between the generations and motivate the inspection of *Wolbachia* infection and strain diversity within the life cycle of *B. treatae*.

### Specimen sampling

We sampled 23 *B. treatae* populations distributed across the range of the three host plants (five *Qf* associated populations, ten *Qv* populations, and  eight *Qg* populations) in Oklahoma, Texas, Mississippi, Alabama, Florida, and Georgia (Fig. 1; Table 1)*.* Asexual adults were obtained from 16 populations by haphazardly collecting between several hundred to several thousand leaf galls from multiple locations within the crowns of trees at each site. Leaf galls were then binned by site in bulk collection traps. Sexual adults were obtained from 15 populations by haphazardly collecting root galls excavated from beneath heavily leaf-galled trees from each site. Root galls were returned to the laboratory and housed individually in standard fruit fly rearing vials. Galls of both generations were maintained outdoors at the Texas State Greenhouse in a shaded alcove under natural conditions. Collections were monitored daily and upon emergence, live adults were immediately stored in 95% ethanol. Details of population locations, host plant affiliation, generation(s) sampled, and sample sizes are shown in Table 1. Genomic DNA was then extracted from whole adult bodies using DNeasy Blood and Tissue kits (Qiagen Inc., Valencia, CA).

**Table 1:** Sample frame for the study of *Wolbachia* infection dynamics in *Belonocnema treatae*

							*Wolbachia* infection prevalence							
#	Site location	Latitude	Longitude	Host	Gen.	n	not inf.	*w*Tre 1	*w*Tre 2	*w*Tre 3	*w*Tre 4	*w*Tre1&3	*w*Tre2&3	*w*Tre1&2&3
1	Quartz Mountains, OK	34.890075	−99.301092	*Qf*	A,S	40	1.00	0	0	0	0	0	0	0
2	Irion, TX	31.214739	−100.842089	*Qf*	A	9	0.89	0.11	0	0	0	0	0	0
3	San Marcos, TX	29.937306	−98.009944	*Qf*	A	20	0.35	0.65	0	0	0	0	0	0
4	Luling, TX	29.680778	−97.650750	*Qv*	A	10	0	0.90	0	0	0	0.10	0	0
5	Encino, TX	26.894167	−98.135194	*Qf*	A,S	50	0	1.00	0	0	0	0	0	0
6	Live Oak Park, TX	27.854383	−97.210494	*Qf*	A,S	46	0	1.00	0	0	0	0	0	0
7	Rice University, TX	29.717389	−95.402278	*Qv*	A,S	30	1.00	0	0	0	0	0	0	0
8	Picayune, MS	30.527103	−89.681217	*Qv*	S	15	0.13	0.87	0	0	0	0	0	0
9	Gautier, MS	30.380386	−88.610331	*Qv*	S	28	0	0.14	0.86	0	0	0	0	0
10	Gulf Shores, AL	30.255450	−87.720475	*Qg*	A,S	20	0.05	0	0.95	0	0	0	0	0
11	Inlet Beach, FL	30.274314	−86.003869	*Qg*	A,S	19	1.00	0	0	0	0	0	0	0
12	Parker, FL	30.112389	−85.603556	*Qg*	A	10	0.90	0	0	0.10	0	0	0	0
13	Lanark Village, FL	29.888431	−84.577019	*Qg*	S	16	1.00	0	0	0	0	0	0	0
14	Ochlockonee, FL	29.960083	−84.385111	*Qg*	A	10	1.00	0	0	0	0	0	0	0
15	Perry, FL	30.116100	−83.589542	*Qv*	A,S	30	0.93	0	0	0	0.07	0	0	0
16	Sapelo Island, GA	31.397528	−81.278631	*Qv*	A	10	0	0	0	0	0	0	1.00	0
17	High Springs, FL	29.843750	−82.631894	*Qv*	S	3	0	0	1.00	0	0	0	0	0
18	Progress Park, FL	29.781222	−82.473361	*Qv*	A	10	0.30	0	0.10	0.60	0	0	0	0
19	Cedar Key, FL	29.150642	−83.047694	*Qg*	S	11	0	0	0.45	0.55	0	0	0	0
20	Lake Lizzie, FL	28.227672	−81.179989	*Qg*	A,S	30	1.00	0	0	0	0	0	0	0
21	Hickory Hammock, FL	27.377917	−81.096889	*Qv*	A	6	0	0	0	0	0	0	0	1.00
22	Kissimmee River, FL	27.377911	−81.096886	*Qv*	S	20	0	0	0	0	0	0.05	0	0.95
23	Dickinson St. Park, FL	27.026194	−80.109161	*Qg*	S	20	0	0	0	1.00	0	0	0	0

*Host* host plant of *B. treatae* (*Qf* = *Quercus fusiformis*, *Qv* = *Q. virginiana, Qg* = *Q. geminata*), *gen* generation examined (A = asexual generation; S = sexual generation), *n* sample size; *Wolbachia* infection frequency and strain type detected per population

### *Wolbachia* genotyping

A total of 463 *B. treatae* individuals from the 23 populations were screened for *Wolbachia* using the primers 81F and 691R [40] that target a ~ 600 bp fragment of the *Wolbachia* surface protein *wsp* gene*.* We performed PCR reactions in a total volume of 10 μl using 10× reaction buffer Y (Peqlab, Germany), 800 μM dNTPs, 0.3 μM of each Primer, 0.5 U peqGold Taq DNA polymerase (Peqlab, Germany) and 1 μl of template DNA. All reactions were performed on a 2720 thermal cycler (Applied Biosystems) with the following conditions: 94 °C for 4 min, followed by 34 cycles at 94 °C for 30 s, 55 °C for 1 min, 72 °C for 1 min and a final extension at 72 °C for 10 min. All PCR products were run on a 2% electrophorese gel and visualized by Gel Red nucleic acid gel staining and UV illumination. To exclude false positives and/or negatives, all PCRs were replicated twice to confirm results. All positive samples were then Sanger sequenced by Eurofins MWG Operon (Ebersberg, Germany). Sequences were individually assembled and manually edited using CodonCode Aligner vers. 3.7.1 (CodonCode Corp., Centerville, MA). All sequences were then screened for ambiguous sites by inspecting all raw chromatograms for multiple peaks, to determine if individuals were infected by multiple *Wolbachia* strains.

To segregate alleles of potentially multi-infected individuals we cloned PCR products from nine individuals selected from geographically separated populations that either showed double and or triple peaks indicating the presence of multiple *Wolbachia* strains. Additionally to check for potentially hidden *Wolbachia* strains (e.g. [41]) we cloned eight individuals whose chromatograms showed clear single peaks suggesting the presence of just one *Wolbachia* strain. Molecular cloning was performed by Eurofins MWG Operon (Ebersberg, Germany) using pTZ57R/T vector (Thermo Scientific, USA) and a TOPO TA cloning kit. Eleven to 19 plasmids per individual were Sanger sequenced and the 286 sequences were screened and edited manually and aligned using CodonCode Aligner.

### Mitochondrial genotyping

We further genotyped a 633 bp region of the mitochondrial cytochrome oxidase I (COI) gene of all 463 *B. treatae* individuals. Individuals were sequenced using the barcode primers LCO 1490 and HCO 2198 [42]. PCR reactions were performed in a total volume of 10 μl containing 10× reaction buffer Y (Peqlab, Germany), 800 μM dNTPs, 0.3 μM of each Primer, 0.5 U peqGold Taq DNA polymerase (Peqlab, Germany) and 1 μl template DNA. The thermocycler protocol was: 94 °C for 3 min, followed by 34 cycles at 94 °C for 30 s, 55 °C for 45 s, 72 °C for 45 s and a final extension at 72 °C for 7 min. All PCR products were run on a 2% electrophorese gel, visualized by Gel Red nucleic acid gel staining and then Sanger Sequenced by Eurofins MWG Operon (Ebersberg, Germany). Resulting sequences were edited manually and aligned using CodonCode Aligner.

### Genetic distance and nucleotide diversity within and among *B. treatae* populations

To characterize patterns of genetic variation within and among *B. treatae* populations, we calculated the average genetic distance between and within groups of *B. treatae* (i.e., host plant-affiliated populations, population pairs, between generations and sexes), measured by the number of nucleotide substitutions occurring between two mtDNA haplotypes using the Tamura Nei model [43] determined by the best fitting model option in MEGA. All analyses were calculated in MEGA v. 6.06 [44]. Given the phylogenetic results (see below), COI haplotypes were then grouped for additional comparisons into western (1–8) and eastern populations (10–23) according to their level of sequence divergence and *Wolbachia* infection status. Population 9 (Gautier, Mississippi) was excluded from this specific analysis as it contained a mix of eastern and western *B. treatae* haplotypes and *Wolbachia* strain infections.

### Phylogenetic history of *Wolbachia* and *B. treatae*

To infer the evolutionary history of *Wolbachia* infection in *B. treatae* and to assess whether *Wolbachia* influenced the mitochondrial diversity of its host, we performed a phylogenetic analysis of *B. treatae* populations using the mtDNA sequence data. We followed the phylogenetic methods of Ronquist et al. [45] that performed a similar analysis using the COI gene for > 100 cynipid gall wasp species, including several taxa from North America closely related to the genus *Belonocnema*. All individuals from each of the 23 populations were included*.* Our analysis contained four cynipid outgroup taxa: *Biorhiza pallida* (GenBank accession number: AY368931), *Cynips quercus* (DQ012638), *Trigonaspis mendesi* (DQ012658) and *Neuroterus numismalis* (AY368930) previously published in [45]. Phylogenetic relationships were estimated using Bayesian inference implemented in MrBayes version 3.2.2 [46] following the methods optimized for mtDNA COI analysis of gall formers detailed in [45]. Here, sequence data was modeled using the four by four nucleotide model that estimates stationary state frequencies that integrate across all grouping and ungrouping exchangeability rate parameters [45, 47]. In addition, we used the mixed-rates evolutionary model with a gamma distribution which allows substitution rates to vary under a flat Dirichlet (default) prior. The protein-coding COI gene was divided into two categories, first and second codon positions and third positions, with model parameters and base rate substitutions uncoupled [45, 46]. Default setting was used for all remaining parameters. The model was run four independent times using four Metropolis-coupled chains, interactively, for three-million generations at a time, until the deviation of split frequencies converged on a value < 0.01 (~ 21 million generations). Chains were sampled once every 1000 generations with an initial burn in of 25%. In each analysis, all branch lengths and substitution model parameters possessed potential scale reduction factors between 1.00 and < 1.01. We then constructed a majority rule consensus tree (presented herein) from the post burn in data.

To provide insight into whether *Wolbachia* infections of *B. treatae* populations observed in our study more likely represent deep ancestral associations with the symbiont, or repeated acquisitions of *Wolbachia* by different gall wasp populations we compared the phylogenetic relationships of the four *Wolbachia* strains found in *B. treatae* using the *wsp* gene sequences with representative *Wolbachia* sequences from other insect systems. To form the phylogeny, we used a consensus *wsp* sequence for each of the three major *Wolbachia* strains (*w*Tre1–3) found in *B. treatae*, sequences from related *Wolbachia* strains available from GenBank and included the rare B-group strain *w*Tre4 as an outgroup. Similar to the methods employed for *B. treatae*, we used MrBayes version 3.2.2 [46] to construct the phylogeny with the following exception: we used the GTR + I + G model of sequence evolution adopted from [48] which was run four independent times for 50,000,000 generations until the deviation of split frequencies converged on a value of 0.003.

### Vertical transmission rate of *Wolbachia*

A concurrent study of multiple paternity in *B. treatae* from single pair crosses involving an additional 353 individuals allowed us to examine the vertical transmission rate of *Wolbachia* from adult sexual generation females to their asexual offspring. This study involved the San Marcos, Texas population (population 3) that was characterized by intermediate infection frequencies of the endosymbiont. Infected individuals carried strain *w*Tre1 (see results below). We assayed the *Wolbachia* infection status of 23 sexual generation females and eleven to 16 asexual progeny produced by each female. The frequency of infection of asexuals produced by infected sexual females estimates the vertical transmission rate of the *w*Tre1 *Wolbachia* strain during this phase of the *B. treatae* life cycle.

### Statistical analysis

We tested for heterogeneity in the frequency of infection among *B. treatae* populations and the influence of host plant association, geography and cynipid generation on the average frequency of *Wolbachia* infection. To test for differences in *Wolbachia* infection among populations we used χ^2^ tests. We first tested for variation among all populations and then subsetting the data we tested for variation among populations within alternative generations (asexual versus sexual), geography/phylogenetic clades (east versus west clade), and host plant association (*Qv*, *Qg*, *Qf*). Given multiple testing, we adjusted our alpha value by the number of tests run from the same dataset to control for Type 1 error (α = 0.05/4 = 0.0125). To test the influence of host plant association and generation on average infection frequency, we ran a t-test or ANOVA on arcsine square root transformed population infection frequencies weighted by the sample size. All statistical analyses were performed in R vers. 3.3.1 [49].

## Results

### *Wolbachia* infections dynamics within and among *B. treatae* populations

**Fig. 2:**
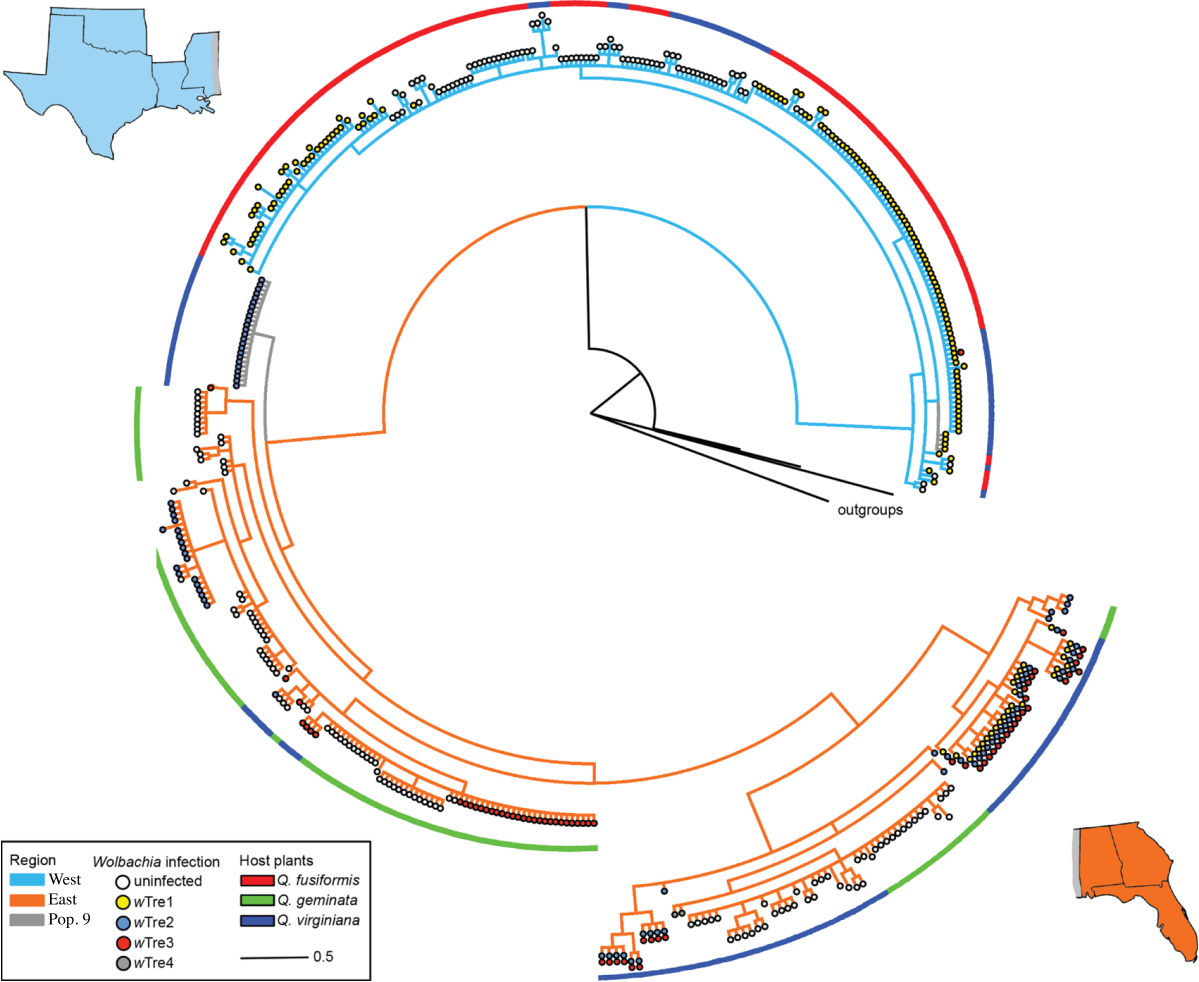
Bayesian phylogenetic tree based on sequencing of the mtDNA COI gene for 463 *Belonocnema treatae* sampled from 23 populations. Branch colors represent geographic separation between populations from West (light blue; populations 1–8) and East (orange; populations 10–23) and population 9 (grey). The outside bar represents the host plant affiliation for each collection site (red = *Q. fusiformis*, green = *Q. geminata* and blue = *Q. virginiana*). The different dots represent the infection status of each single individual with white = uninfected, yellow = *w*Tre1, blue = *w*Tre2, red = *w*Tre3, grey = *w*Tre4. Individuals with two or three dots indicate infection by more than one *Wolbachia* strain

Across all *B. treatae* populations, 56.1% of the 463 individuals screened were infected by *Wolbachia*. Infection frequency varied markedly among the 23 populations (*X*^2^ = 409, df = 22, *P* < 0.0001; Fig. 1). We found 100% infection in ten populations and 0% infection in six populations (Table 1). In seven populations, both infected and uninfected individuals were present (Table 1), with infection frequencies ranging from 11% to 95%. When *B. treatae* populations were grouped into western and eastern clades based on mtDNA haplotypes (see below), the *Wolbachia* infection frequency differed among populations within both the western clade (*X*^2^ = 190, df = 7, *P* < 0.0001) and the eastern clade (*X*^2^ = 190.57, df = 13, *P* < 0.0001). There was no association between the *Wolbachia* infection status (infected or uninfected) and mtDNA haplotype (*X*^2^ = 0.01, df = 91, *P* = 0.9591; Fig. 2).

We also tested for variation in *Wolbachia* infection frequency across *B. treatae* populations affiliated with each of the three host plant species. We found *Wolbachia* infection frequency to be 37.5% for individuals sampled from *Qg* (*N* = 136), 61.1% for individuals sampled from *Qv* (*N* = 162), and 66.7% for individuals from *Qf* (*N* = 165). However, within each host plant association, infection frequencies among populations were highly variable (*Qg*: *X*^2^ = 129.1, df = 7, *P* < 0.0001; *Qf*: *X*^2^ = 149.8, df = 4 *P* < 0.0001; *Qv*: *X*^2^ = 127.6, df = 9, *P* < 0.0001). ANOVA of population infection frequencies weighted by sample size showed no evidence of statistically significant differences in infection frequencies among the three host plants (F_2,20_ = 0.54, *P* = 0.5910). Notably, we found populations with 100%, 0%, and partial infection frequencies associated with all three host plants.

Comparison of the frequency of *Wolbachia* infection between the generations across all populations showed no general difference between asexual (0.53 ± 0.11 SE) and sexual (0.62 ± 0.09 SE) individuals (*t* = 0.75, df = 29, *P* = 0.4574). Moreover, the eight sites containing samples from both sexual and asexual generations provided a direct comparison of infection rates among the alternate generations while excluding the effects of variation in infection frequencies among populations (Additional file 1: Table S1). This direct comparison demonstrated near identical *Wolbachia* infection frequencies between the sexual and asexual generations of *B. treatae* (paired t-test: *t* = 1.23, df = 7, *P* = 0.2583).

### High mitochondrial diversity within *B. treatae*

Characterization of the 633 bp fragment of the mtDNA COI gene of 463 *B. treatae* individuals sampled from 23 populations distributed across the southern U.S. yielded 92 distinct haplotypes (GenBank Accession Numbers MG252379–252470). Haplotypes clustered into two major clades: a western clade encompassing populations from Oklahoma, Texas and Mississippi (populations 1–8) and an eastern clade encompassing populations from Florida, Georgia and Alabama (populations 10–23) (Fig. 2). Population 9, from Gautier, Mississippi, geographically located between populations grouping with the eastern and western clades, contained individuals with haplotypes from both the eastern and the western clades (Figs. 1 and 2).

Sequence divergence between individual mtDNA haplotypes ranged from 0.2% to 7.2% indicating considerable variation in the degree of divergence among the 23 *B. treatae* populations. The genetic distance between western and eastern clades was 6.3%, while the mean genetic distance between pairs of populations that clustered within the western and eastern clades was 0.5% and 2.1%, respectively (Table 2). Within each of the three sets of host plant-affiliated populations the mean genetic distance between pairs of populations was 0.4% in *Qf*, 3.8% in *Qv* and 1.6% in *Qg* indicating greater among population genetic distance among populations resident on *Qv.* When the ten *B. treatae* populations that used *Qv* as the host plant were binned into eastern and western clades, the mean genetic distance between pairs of western populations of *B. treatae* on *Qv* was 0.4% compared to 2.2% between pairs of eastern populations (Table 2). Finally, the average pairwise genetic distance among sexual (0.4%) and asexual (0.5%) populations in the western clade was lower than corresponding observed average genetic distances in the eastern clade (2.1% sexual generation and 2% in the asexual generation). These generation-specific estimates of genetic distance among populations accord with the average genetic distance estimates among pairs of populations clustering in the western and eastern clades (above) and suggest no influence of asexual versus sexual reproduction on sequence divergence (Table 2).

**Table 2:** Summary of sequence diversity of the mitochondrial COI gene within and between *Belonocnema treatae* populations

Sequence diversity (within populations)				Sequence divergence (between populations)			
	n ind	n ht	d		n ind	n ht	d
East	239	54	0.021	West x East	463	92	0.063
West	224	38	0.005	Infected x uninfected West	224	38	0.005
Infected West	137	21	0.004	Infected x uninfected East	239	54	0.022
Uninfected West	87	19	0.003	*Qf* x *Qv* West	224	38	0.005
Infected East	123	31	0.020	*Qf x Qv* East	268	58	0.063
Uninfected East	116	26	0.020	*Qf* x *Qg*	301	52	0.063
*Qg*	136	25	0.016	*Qg* x *Qv* West	195	41	0.062
*Qf*	165	27	0.004	*Qg* x *Qv* East	239	56	0.024
*Qv*	162	47	0.038	*Qv* West x *Qv* East	162	47	0.062
*Qv* East	103	31	0.022	West sex x West asex	224	38	0.005
*Qv* West	59	16	0.004	West sex x East sex	273	60	0.063
Sex West	119	26	0.004	West sex x East asex	204	53	0.062
Asex West	105	24	0.005	West asex x East sex	259	58	0.063
Sex East	154	34	0.021	West asex x East asex	190	51	0.063
Asex East	85	27	0.020	East sex x East asex	239	54	0.021

East and West denote the eastern and western *B. treatae* clades based on phylogenetic analysis of the *B. treatae* populations

*n ind* number individuals, *n ht* number of haplotypes, *d* average evolutionary distance over sequence pairs between and within groups

### *Wolbachia* strain diversity and co-occurrence in *B treatae* populations

By sequencing the *wsp* gene of all 260 *Wolbachia* infected individuals and 286 clones derived from 17 individuals, we found that *B. treatae* is infected by four *Wolbachia* strains designated *w*Tre1–4 (GenBank accession numbers MG252471–252474). Strain *w*Tre1 was mainly present in the western *B. treatae* mitochondrial clade, which included populations from Oklahoma, Texas and western Mississippi. In this clade, all infected individuals harbored only *w*Tre1, with the exception of a single individual sampled in Luling, Texas, confirmed by TOPO cloning to be double infected by *w*Tre1&3 (Fig. 1). The three other *Wolbachia* strains were found exclusively in the eastern *B. treatae* clade. Strain *w*Tre2 was present in varying frequencies in populations sampled in Mississippi, Alabama and northern Florida while individuals from Gautier, Mississippi (population 9), were either infected by *w*Tre1 or by *w*Tre2. Strain *w*Tre3 was present in two populations in northern Florida and one population in southern Florida, while *w*Tre4 was detected in only two individuals from Perry, Florida (population 15). All *B. treatae* individuals from Sapelo Island, Georgia (population 16) were confirmed to be double infected by *w*Tre2&3 and all but one individual collected at Hickory Hammock, Florida (population 21) and Kissimmee River, Florida (population 22) was triple infected by *w*Tre1&2&3, with a single individual found to harbor strains *w*Tre1 and *w*Tre3 only (Fig. 1).

**Fig. 3:**
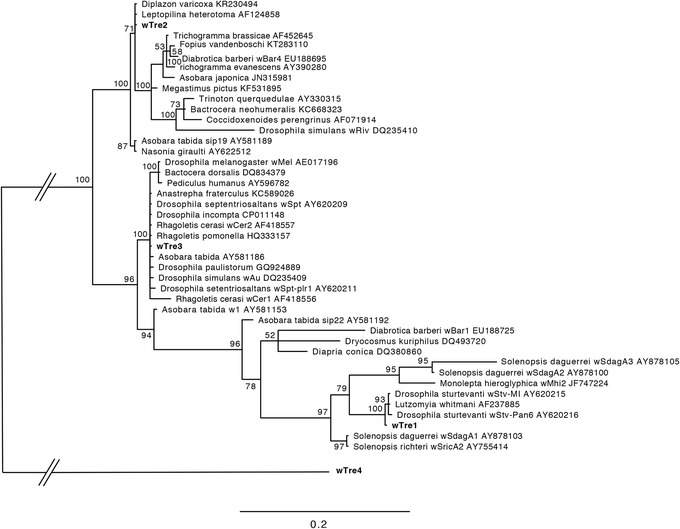
Bayesian phylogenetic tree based on *wsp* surface protein gene sequences for the three most common *Wolbachia* strains *w*Tre1, *w*Tre2 and *w*Tre3 from supergroups A and a sample of related *Wolbachia* sequences from insect taxa obtained from GenBank. We used the rare B-group strain *w*Tre4 as outgroup. Bootstrap values above 50% are shown. The branch between supergroup A and supergroup B was reduced as indicated by double-bars

The phylogeny of the *wsp* gene shows that the three major *Wolbachia* strains (*w*Tre1–3) detected in *B. treatae* belong to the *Wolbachia* supergroup A. Strain *w*Tre4, however, belongs to the supergroup B (Fig. 3). Within the A-group, the three *B. treatae* associated strains were more closely related to *Wolbachia* strains from other species and geographic localities than they were to each other (Fig. 3). This pattern is consistent with *B. treatae* acquiring multiple infections through time.

### *Wolbachia* and haplotype diversity of *B. treatae*

There was no strict association between *B. treatae* mtDNA haplotype and *Wolbachia* infection status across the range of the insect host (Fig. 2). However, inspection of *B. treatae* populations that contained both infected and uninfected individuals or individuals infected by different *Wolbachia* strains suggest effects of the endosymbiont on haplotype diversity of its host. For example, mitochondrial haplotypes that clustered either into the western or eastern clade were associated with their *Wolbachia* infection. Specifically, *w*Tre1 infected individuals were exclusively associated with western haplotypes and *w*Tre2 infected individuals were exclusively associated with eastern haplotypes (Fig. 2). Although there was no strict range-wide association of *Wolbachia* strain and mtDNA haplotype, two of the 23 populations did exhibit such patterns: Individuals from Gautier, Mississippi (population 9), were either infected by *w*Tre1 or by *w*Tre2, where *w*Tre1 was associated directly with a western mtDNA haplotype and *w*Tre2 was associated with an eastern mtDNA haplotype. We found a similar pattern in Cedar Key, Florida (population 19), where individuals were either infected by *w*Tre2 or *w*Tre3. The mtDNA sequence diversity within *w*Tre2 infected hosts was 0.3% while all *w*Tre3 infected gall wasps shared the same haplotype. In contrast, the sequence divergence between individuals infected by the two different *Wolbachia* strains was 2.6% which is consistent with a strict association of *Wolbachia* with specific mitochondrial haplotypes at the population level. In the population from Progress Park, Florida (population 18), we found individuals that were uninfected, one individual infected by *w*Tre2 and individuals infected by *w*Tre3 (Fig. 1). While the *w*Tre2 infected and uninfected individuals shared the same haplotype, the *w*Tre3 infected gall wasps exhibited different mtDNA haplotypes with a sequence divergence of 0.5%.

The strain *w*Tre1 was present in *B. treatae* developing on both plant hosts, *Qf* and *Qv,* in the west. Similarly, *w*Tre2 and *w*Tre3 were detected in *B. treatae* developing on *Qg* and *Qv* in the east. The double and triple infection in the eastern clade, however, occurred exclusively on individuals attacking *Qv.* The three strains occurred in both asexual and sexual *B. treatae* generations while *w*Tre4 was detected exclusively in two sexual females from Perry, Florida (population 15).

In addition to identifying the four *Wolbachia* strains, sequencing of 286 clones from 17 individuals indicated the presence of widely dispersed SNPs across the 600 base pair fragment of the *wsp* gene. High SNP diversity was found in *w*Tre1 (100 SNPs in 126 clones), *w*Tre2 (54 SNPs in 67 clones) and *w*Tre3 (78 SNPs in 93 clones). Of the total of 232 SNPs detected, 96% were singletons and just ten SNPs were confirmed by independent PCRs. Although we cannot rule out occasional sequencing and cloning errors, the high incidence of SNPs detected within strains suggests that it is likely that different subtypes of the four main strains are present.

### *Wolbachia* transmission frequency between generations

Of the 23 sexual females examined to assess transmission of *Wolbachia* to their asexual progeny, ten females were positive for *Wolbachia* while 13 were not infected by the endosymbiont. Within infected females in three cases, 100% of the offspring (*N* = 14–16 per female) were also infected by the endosymbiont suggesting perfect maternal transmission (Additional file 2: Table S2). However, in seven cases, *Wolbachia* transmission was imperfect with transmission rates ranging from 64% to 94%. The average transmission rate estimated across the ten females was 87.5% (Additional file 2: Table S2). Further, 182 progeny (*N* = 14 per female) of the 13 females not infected by *Wolbachia* were additionally tested. For all 13 females, 100% of their offspring was also tested negative (Additional file 2: Table S2).

## Discussion

We examined a widely distributed North American species of cynipid gall forming wasp that exhibits regional host specialization and asked whether patterns of *Wolbachia* infection and diversity are associated with the insect’s host plant association, geographic distribution, mitochondrial evolutionary history, and life cycle. By analyzing *Wolbachia* infection status and strain diversity within and among individual gall formers drawn from 23 *B. treatae* populations distributed across the three host plant species sampled from across the southern U.S., we demonstrated highly variable *Wolbachia* infection and high strain diversity among populations. We found little evidence that host plant affiliation of the gall former was associated with *Wolbachia* infection status, however we did find evidence of an association between *Wolbachia* strain and geography of the host. Finally, we detected *Wolbachia* in equal frequencies in both asexual and sexual generations and between males and females of the sexual generation. This conforms with previous studies that suggest that *Wolbachia* may not influence the sex determination system in gall wasps [26, 29, 30].

### Diversity and distribution of *Wolbachia* in divergent *B. treatae* lineages

In contrast to patterns predicted by selective sweeps of mtDNA haplotypes associated with *Wolbachia* induced CI [11, 15, 50, 51], we detected no difference in mitochondrial haplotype diversity between *Wolbachia* infected and uninfected populations of *B. treatae.* However, we did find strong associations between *Wolbachia* strain type and the geographic distribution of *B. treatae*: strain *w*Tre1 is almost exclusively present in populations from the western portion of the geographic range of *B. treatae*, while strains *w*Tre2 and *w*Tre3 are almost exclusively present in populations from the east. Strain *w*Tre4 was a low frequency strain found only in two individuals in one population from Florida (Fig. 1). This pattern of distribution of *Wolbachia* strain types was matched by the significant break in the mtDNA gene tree between eastern and western *B. treatae* populations (Fig. 2).

Two alternative scenarios could explain the observed distribution patterns of the three most frequent strains, *w*Tre1–3. First, *Wolbachia* infection in *B. treatae* may be an ancient infection present before the divergence of *B. treatae* into eastern and western mtDNA clades. In this scenario, the three major *Wolbachia* strains have evolved following the geographic separation of the host. While, secondary endosymbionts like *Wolbachia* are able to cross species boundaries and can therefore be randomly distributed among different hosts [52, 53] coevolution is also possible where *Wolbachia* can persist in host lineages more than a million years [54]. Alternatively, *B. treatae* populations may have acquired *Wolbachia* strains independently after the divergence of eastern and western mtDNA clades. The fact that different *w*Tre strains do not form a monophyletic clade but rather are distributed across the *Wolbachia* phylogenetic tree and are related to strains found in other insect hosts (Fig. 3), suggests that *B. treatae* populations have independently acquired different *Wolbachia* strains after separation into eastern and western clades. This scenario also conforms with the observation that distinct eastern and western mitochondrial haplotypes are co-associated with the same *Wolbachia* strain, further suggesting that independent horizontal transmissions of *Wolbachia* could have produced this pattern. Under this scenario *B. treatae* populations that are not infected by *Wolbachia* simply reflect populations that have not yet been invaded by the endosymbiont. However, as supported by our findings of incomplete vertical transmission of the symbiont, these uninfected populations may also have lost their associated *w*Tre strains. Within well studied populations of live oaks a minority of individual live oak trees that are patchily distributed typically support the vast majority of *B. treatae* [55]. Further these infrequent trees harboring *B. treatae* experience orders-of-magnitude among-year-variation in the abundance of *B. treatae*, (personal observation). Together, these features of the ecology of *B. treatae* in concert with incomplete vertical transmission may set the stage for periodic loss of infections.

To resolve if a *Wolbachia* driven selective sweep has influenced the mitochondrial structure of its host, future studies need to examine nuclear genetic markers. While a disconcordant pattern of mitochondrial and nuclear DNA would support the hypothesis of a *Wolbachia*-driven selective sweep, a concordant pattern would suggest that other factors such as the demographic history of the host have driven the observed mitochondrial divergence [56]. Moreover the microbial community of *B. treatae* needs to be characterized more comprehensively to understand if any other symbiont might have influenced the observed pattern.

### *B. treatae* populations harbor multiple *Wolbachia* strains

Two intriguing aspects of our results are the detection of multiple *Wolbachia* strains within five of the 23 surveyed *B. treatae* populations and the presence of multiple strains within individual gall formers. Study-wide, among the 260 individuals infected with *Wolbachia*, we found that 86% of individuals harbored a single *Wolbachia* strain. In population 9 in Gautier, Mississippi, we found individual *B. treatae* that were infected with *w*Tre1 or *w*Tre2 but none of the 28 individuals examined were infected by both strains. Similarly, individuals from populations from Cedar Key, Florida (population 19), and Progress Park, Florida (population 18), were infected by either *w*Tre2 or *w*Tre3 but no individual in these populations were simultaneously infected by both strains. In contrast, these same two strains occur as a double infection in all ten individuals sampled from Sapelo Island, Georgia (population 16), and all three major *w*Tre strains occur within the same individuals from central Florida (Hickory Hammock [population 21] and Kissimmee River [population 22]; Fig. 1; Table 1).

The presence of multiple *Wolbachia* strains within single individuals has been documented in several species [41, 57–59] including three gall wasp species [30]. In such cases the horizontal acquisition of a new strain [12] or a spreading infection from a restricted host population [11] can cause host populations to acquire additional strains. Other cases, however, suggest that a spreading new *Wolbachia* strain is displacing a previously present infection [10]. Lastly, theoretical models have shown that spatial structure and habitat fragmentation can promote the coexistence of *Wolbachia* [60], however the general mechanisms whereby individual insects acquire and maintain multiple strains are not clearly understood and requires further work.

Most of the double and all of the triple infected individuals that we detected clustered together on one branch of the mtDNA tree (Fig. 2) along with some uninfected and single infected individuals. All of the single infected individuals on this branch are infected by *w*Tre2 suggesting that the double infections present in all individuals in Sapelo Island, Georgia (population 16) might have resulted from a horizontal acquisition of *w*Tre3 by previously *w*Tre2 single-infected individuals. Similarly, in the triple infected populations from central Florida, previously *w*Tre2 infected individuals might have acquired both *w*Tre1 and *w*Tre3 horizontally. It is unclear why the *w*Tre1 infection that occurs in multiple western populations as the only strain, appears as a triple infection in central Florida.

Parasitoids and inquilines are possible sources of horizontal *Wolbachia* transmission [61–63]. *Belonocnema treatae* is attacked by a diverse natural enemy community composed of parasitoids and inquilines [32], of which some species are known to harbor *Wolbachia* infections e.g. [64, 65]. This natural enemy community represents an extensive source of potential transmitters of *Wolbachia* strains. More broadly the three species of live oaks that are galled by *B. treatea* are also the host plants of a diverse community of cynipid gall formers. The shared habitat of communities of gallers and their associated natural enemies [24, 66, 67] represents a complementary avenue of direct or indirect *Wolbachia* exchange. Thus, a possible explanation for the occurrence of *w*Tre1 in the eastern clade is horizontal transmission of *Wolbachia* by parasitoids, inquilines, or other gall wasp species co-occurring on the same host plants. Studies of communities of gall wasp species and their respective communities of parasitoids and inquilines associated with shared host plant species e.g. [57] are needed to elucidate the transmission and subsequent population dynamics of single and multiple strains of *Wolbachia* within and among trophic levels for these insect communities. Additionally, studies that examine the relationship between variation in parasitoid pressure (i.e., rate of parasitism and diversity of parasitoids) among *B. treatae* populations with different *Wolbachia* infections are needed to understand the distribution of *Wolbachia* in *B. treatae*.

An alternative explanation of the occurrence of *w*Tre1 in eastern populations (Fig. 1) is that the eastern and the western *w*Tre1 infections actually constitute different *Wolbachia* strains that share the same *wsp* alleles and hence are not distinguishable by our study. Additional multilocus sequence typing (MLST) of five conservative genes can resolve hidden genetic diversity of *Wolbachia* [68]. To gain more insight into the evolutionary history of *w*Tre strains in *B. treatae* a future study will focus on a more extensive genomic characterization of the four *w*Tre strains detected in this host species to resolve potential co-evolution of the endosymbiont with its host. However, delineation of closely related strains can still be challenging when using MLST [69, 70]. Thus, in addition to characterizing additional MLST genes, a comparative genomic approach involving sequencing of individual *B. treatae* and *Wolbachia* from diverse populations will aid in confirming both the relationships among the putatively different *w*Tre strains and delineating population substructure of the host.

### Potential role of *Wolbachia* in reproductive isolation of *B. treatae*

The potential role of *Wolbachia* as a driving factor in insect speciation remains controversial [2, 6, 7, 18]. *Wolbachia* infection needs to fulfill two conditions to cause speciation: 1) infection polymorphisms among different host populations must be stable and 2) cytoplasmic incompatibility must be strong enough to generate natural selection favoring the evolution of reproductive isolation which promotes divergence of its host [2]. Unidirectional CI appears insufficient as it results in unstable equilibrium frequencies and therefore the spread of the bacterium is predicted to occur more rapidly than the evolution of reproductive isolation [9, 20]. Importantly however, theoretical predictions show that two bidirectional CI-inducing *Wolbachia* strains can be sufficiently stable and demonstrate that gene flow can be substantially reduced between hosts harboring different strains [71, 72]. However, few studies have demonstrated coexistence of different *Wolbachia* strains along a contact zone [73]. In our study, while most infected *B. treatae* populations are infected by a single *Wolbachia* strain we also found three populations containing individuals multiply-infected by different *w*Tre strains. The occurrence of different *Wolbachia* strains within *B. treatae* populations highlights the potential of *Wolbachia* to contribute to the diversification in this gall wasp. In particular, the strict association of *Wolbachia* with the mitochondrial haplotype of the individuals in Gautier, Mississippi, and Cedar Key, Florida, where the *Wolbachia* infection status of all 39 individuals corresponds to a specific haplotype, further suggests a potential role of *Wolbachia* in reproductive isolation with either no hybridization between gall wasps infected by different *w*Tre strains or perfect CI with complete maternal transmission of *Wolbachia*. A preliminary analysis of population genetic structure of *B. treatae* throughout its geographic range based on over 41,000 SNP loci supports the presence of two independent *B. treatae* lineages in the Gautier population (unpublished data). Future studies of the dynamics of *Wolbachia* strains in *B. treatae* should include characterizing the phenotypic effects of the different *w*Tre strains, further exploration of populations with two or more strains, and detailed crossing studies to address CI directly. If these strains are shown to cause CI, the co-occurrence of individuals infected by different strains could lead to bidirectional CI. In this case *Wolbachia* could act as a potential factor in driving reproductive isolation of its host e.g. [19]. Detailed inspection of the populations in Gautier, Mississippi, where *w*Tre1 and *w*Tre2 infected *B. treatae* individuals meet in a population that may represent a zone of secondary contact, and populations in Cedar Key, Florida, where *w*Tre2 and *w*Tre3 infected individuals co-occur, will show to what degree the two strains might cause CI and influence pre- and postzygotic isolation in *B. treatae* [22, 23]. Finally, crossing studies should investigate the maternal transmission efficiencies of the four *Wolbachia* strains as a means of explaining the absence of the endosymbionts in populations containing uninfected individuals.

## Conclusion

In this study, we surveyed for the presence and diversity of *Wolbachia* in the heterogonic gall wasp *B. treatae*. We did not detect significant differences in the frequency of *Wolbachia* infection among populations of gall wasps attacking different host plants or among the sexual and asexual generation. Rather the distribution of the three common *Wolbachia* strains – with *w*Tre1 present exclusively in the western clade and *w*Tre2 and *w*Tre3 (that with the exception of a single individual) being exclusively present in gall wasps from the eastern clade – highlights the role of geography in the *Wolbachia* infection status of this gall wasp species. The occurrence of different *Wolbachia* strains within *B. treatae* populations and their strict association with mitochondrial haplotypes of the host suggests a potential role of the endosymbiont in reproductive isolation in *B. treatae*.

## Additional files

Additional file 1: **Table S1.** Infection frequencies of *Wolbachia* strains in *B. treatae* populations by host plant, generation and sex. Host = host plant of *B. treatae*, Gen = generation examined, Sex = female (f), male (m), n = sample size and *Wolbachia* infection. (XLSX 23 kb)

Additional file 2: **Table S2.** Maternal transmission rate of *Wolbachia*. Infection = infection status of the female, offspring = number of offspring tested for *Wolbachia*, uninfected = number of uninfected individuals, infected = number of infected individuals, transmission rate = transmission rate of *Wolbachia* from infected mothers to their offspring. (XLSX 42 kb)

## Abbreviations

CI: Cytoplasmic incompatibility
COI: Cytochrome oxidase I
MLST: Multilocus sequence typing
mtDNA: Mitochondrial DNA
*Qf*: *Quercus fusiformis*
*Qg*: *Quercus geminata*
*Qv*: *Quercus virginiana*
SE: Standard error
*wsp*: *Wolbachia surface protein*
*w*Tre: *Wolbachia* from *B. treatae*
